# Immunotherapy response in microsatellite-stable poorly differentiated thyroid carcinoma with mismatch repair deficiency and high tumor mutational burden

**DOI:** 10.20945/2359-4292-2026-0008

**Published:** 2026-01-28

**Authors:** João Henrique Feldmann, João Felipe Feldmann, Cassio Murilo Hidalgo-Filho, Gustavo Luis Contado Alves, Rafael Sarlo Vilela, Sérgio Gonçalves, Gilberto de Castro Júnior

**Affiliations:** 1 Departamento de Oncologia Clínica, Hospital Sírio-Libanês, São Paulo, SP, Brasil; 2 Departamento de Patologia, Hospital Sírio-Libanês, São Paulo, SP, Brasil; 3 Departamento de Cirurgia de Cabeça e Pescoço, Hospital Sírio-Libanês, São Paulo, SP, Brasil

**Keywords:** Thyroid cancer, poorly differentiated thyroid cancer, immunotherapy, MSH2, microsatellite stable

## Abstract

Poorly differentiated thyroid carcinoma (PDTC) is a rare and aggressive malignancy with a poor prognosis. Immunotherapy is typically guided by agnostic biomarkers such as microsatellite instability-high or high tumor mutational burden (TMB); however, these biomarkers are uncommon in PDTC. Therefore, identifying alternative predictive biomarkers remains an urgent necessity. We report the case of a 71-year-old woman who presented with life-threatening locoregional disease and was ineligible for radioiodine or tyrosine kinase inhibitors due to a prior subarachnoid hemorrhage. Molecular profiling of the resected tumor revealed a high TMB (10 mut/Mb), somatic mutations in MSH2 and ATM, and microsatellite stability (MSS). Immunohistochemistry demonstrated complete loss of MSH2/MSH6 expression, while PD-L1 expression was 20% (tumor proportion score). Based on these findings, pembrolizumab was initiated as first-line therapy. The patient experienced clinical improvement and maintained a sustained partial response for seven months, with excellent tolerability. This case represents one of the few documented reports of PDTC with MSS exhibiting marked responsiveness to immunotherapy. Our findings underscore that alternative biomarkers, such as somatic mutations in DNA repair genes including MSH2 and ATM, may predict unexpected responses to immune checkpoint blockade and inform therapeutic decisions, even in the context of MSS and borderline TMB. Broader implementation of molecular profiling is warranted to identify such patients.

## INTRODUCTION

Thyroid cancer (TC), the most prevalent endocrine malignancy, has experienced a 313% surge in global incidence over the past four decades. In the United States alone, 44,020 new cases are projected for 2025, with over 87% classified as well-differentiated thyroid carcinomas (WDTCs) ^(1)^. Poorly differentiated thyroid carcinoma (PDTC) accounts for only 2–15% of cases but contributes disproportionately to TC-related mortality. Originating from follicular cells, PDTC represents an intermediate clinicopathological entity between WDTC and anaplastic thyroid carcinoma (ATC) and is currently recognized as a distinct disease category by the World Health Organization. Although only 15% of PDTC patients present with metastases at diagnosis, distant metastatic disease develops in 37–85% of cases, and 60% of patients succumb to disease progression. Survival rates decline significantly, from 50–85% at 5 years, to 34–50% at 10 years, and ultimately to 0% at 15 years ^(2)^.

The spectrum of genomic alterations in PDTC reflects a highly heterogeneous disease, mainly due to varying diagnostic criteria employed across studies ^(3)^. The molecular profile is characterized by mutations in *HRAS*, *KRAS*, and *NRAS* (25–35% combined), *BRAF* (15–33%), components of the PI3K/AKT/mTOR pathway (11%), *PTEN* (4–33%), *EIF1AX* (11%), *TERT* promoter (40%), *TP53* (16–28%), and *ATM* (7%), resulting in an intermediate mutational profile between WDTC and ATC ^(4,5)^. Compared to ATC, PDTC exhibits a higher frequency of gene fusions (10–14%) ^(5)^.

Regarding tumor mutational burden (TMB), PDTCs have a reported median of 2 mutations per megabase, and programmed death-ligand 1 (PD-L1) expression levels are similarly low ^(6)^. Although PDTC is not typically associated with classic biomarkers predictive of response to immunotherapy, the tumor microenvironment (TME) remains dynamic, with immune editing and tumor-cell interactions influencing cytotoxic or immunosuppressive phenotypes ^(6)^. Given the limited treatment options and absence of established therapeutic targets, next-generation sequencing (NGS) panels, as recommended by European Society for Medical Oncology guidelines, are essential ^(7)^.

Here, we present an exceptionally rare case of metastatic PDTC showing a remarkable and durable response to immunotherapy in a context traditionally considered unfavorable for such treatment due to microsatellite stability (MSS), borderline TMB, and urgent need for rapid therapeutic response given the risk of airway obstruction. To our knowledge, this is among the first reported cases to suggest that additional defects in DNA repair pathways, such as MSH2 and ATM mutations identified in our patient, may represent actionable biomarkers to guide clinical decision-making, with outcomes surpassing those observed in studies supporting biomarker-agnostic use of immunotherapy.

## CASE REPORT

**Figure 1:**
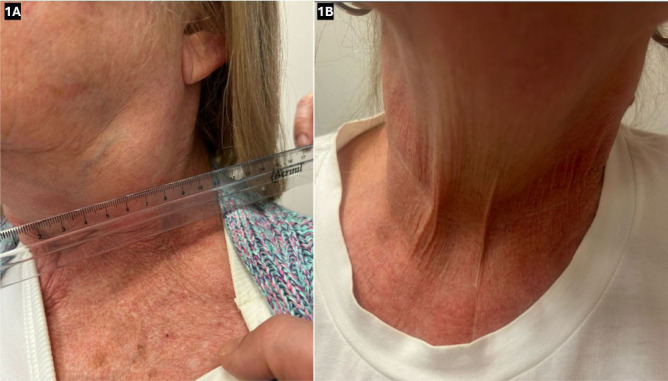
Rapid and marked early clinical response to pembrolizumab. A) Left-sided cervical mass, approximately 8 cm in its greatest dimension, noted to be fixed and firm upon palpation. B) Palpation and visual assessment of the left cervical mass showed marked tumor reduction following three cycles of pembrolizumab.

A 71-year-old self-identified White, Hispanic/Latina female with no significant medical, psychosocial, or family history presented with a painless, progressively enlarging left cervical mass over three months (Figure 1A). Doppler ultrasound of the thyroid revealed a solid, expansive lesion in the left thyroid lobe and left cervical lymphadenopathy at levels IIA and IV. Contrast-enhanced computed tomography of the neck and chest demonstrated extensive local invasion, including tracheal narrowing, esophageal compression, obliteration of the left retropharyngeal space, and approximately 180° encasement of the left common carotid artery. The lesion measured 13.7 × 6.8 × 6.5 cm. Additional suspicious lymph nodes were identified at levels II, III, and the left supraclavicular region. Pulmonary parenchyma appeared unremarkable. Bronchoscopy and nasofibrolaryngoscopy confirmed tracheal stenosis due to extrinsic compression and mild asymmetry of the laryngeal region. Clinical staging, according to the 8^th^ edition of the American Joint Committee on Cancer, was cT4bN1bM0.

**Figure 2:**
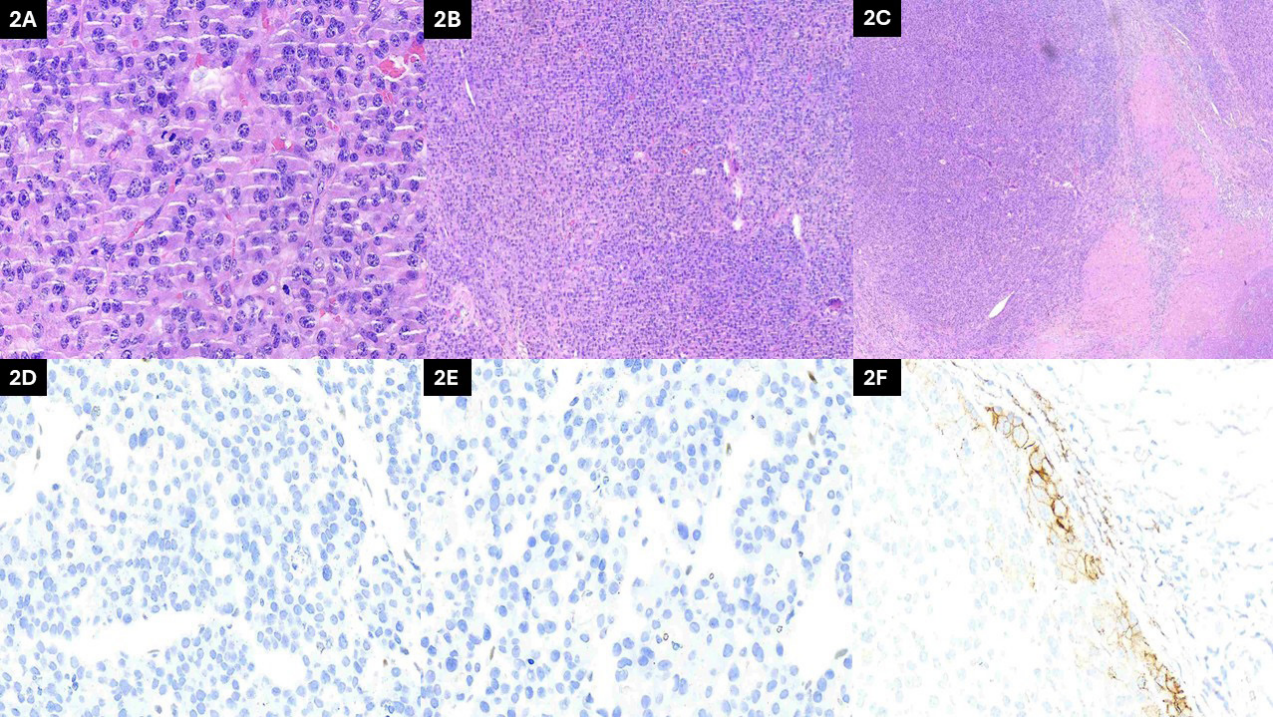
Pathological features of solid carcinoma with PD-L1 expression and MMR deficiency. A) hematoxylin and eosin (H&E), 40× magnification: solid-pattern carcinoma with atypical nuclei and frequent mitoses. B), H&E, 10× magnification: solid neoplasm with convoluted nuclei, irregular chromatin, and occasional nucleoli. C), H&E, 4× magnification: overview of the solid tumor; left side highlights nuclear details; right side indicates extensive necrosis. D), immunohistochemistry (IHC) MSH6 (SP93), 40× magnification: loss of nuclear expression. E) IHC MSH2 (G219-1129), 40× magnification: loss of nuclear expression. F), IHC PD-L1 (SP263), 40× magnification: ~20% tumor proportion score with membranous staining.

Fine needle aspiration of the thyroid lesion and left supraclavicular lymph node revealed a proliferative epithelial lesion. Immunohistochemistry was positive for thyroglobulin, TTF-1, PAX8, and GATA3, and negative for calcitonin, supporting the suspicion of a neoplasm of follicular origin. The patient subsequently underwent total thyroidectomy, parathyroidectomy, and lymph node dissection at levels II–VII. Pathological analysis identified a PDTC, based on the latest World Health Organization pathology criteria, measuring 15 cm, with vascular invasion, multifocal disease, and gross extrathyroidal extension into adjacent tissues (Figures 2A–C). Margins were involved, but sampled lymph nodes were negative for malignancy. In the postoperative period, the patient developed a subarachnoid hemorrhage (Fisher IV) with thoracic subdural extension, resulting in motor, speech, and swallowing deficits. This complication necessitated prolonged hospitalization and delayed oncologic follow-up.

Approximately one year after surgery, fluorodeoxyglucose-labeled positron emission tomography indicated a recurrent expansive lesion occupying the left retropharyngeal space, causing oropharyngeal bulging and partial obliteration of the left piriform sinus. Additional findings included lymphadenopathy at levels Ib and II on the left, with displacement of the trachea and esophagus, a right paratracheal lymph node, and distant fluorodeoxyglucose-avid lesions consistent with pulmonary nodules and a lesion in the left costal arch. Given concerns for systemic recurrence, a new lymph node biopsy was performed, and an NGS-based multigene somatic panel was conducted on the thyroidectomy specimen. This analysis involved DNA sequencing for single and multiple nucleotide variants and small insertions and deletions in 510 genes, as well as RNA sequencing for gene fusions in 55 genes, including detection of oncogenic isoforms and alternative splicing variants in 3 genes. These assays were performed using the Illumina TruSight Oncology 500 panel on the Illumina NextSeq 550 platform, utilizing a custom analysis pipeline within BaseSpace Variant Interpreter (v. 3.6.2.0).

Molecular analysis demonstrated microsatellite stability, a TMB of 10 mut/Mb, which is considered high according to the adopted cutoff of ≥10 mut/Mb, and somatic pathogenic variants in *MSH2* (c.1865del, p.(Pro622GlnfsTer13)) and *ATM* (c.5825C>T, p.(Ala1942Val)). Additional mutations were detected in *BCOR, CIC, DAXX*, *PPM1D, SPEN*, and *TP53*. Immunohistochemistry for DNA repair enzymes showed loss of *MSH2* and *MSH6* expression (Figures 2D–E). PD-L1 expression, assessed using the SP263 clone, revealed a tumor proportion score (TPS) of 20% (Figure 2F). Given the diagnosis of metastatic PDTC with DNA repair deficiency, high TMB, positive PD-L1 expression, and a somatic *MSH2* mutation, the patient was started on tumor-agnostic pembrolizumab monotherapy at a dose of 200 mg every three weeks, consistent with the KEYNOTE-158 study ^(8)^. The patient achieved symptomatic control, along with a partial response of the primary thyroid lesion, as evidenced by considerable reduction in size (Figure 1B), and stable disease in the lymph nodes, lungs, and bones. Treatment was well tolerated, with immune-mediated hypophysitis and adrenal axis deficiency as the only observed immune-related toxicities; both were managed with physiological doses of glucocorticoids and mineralocorticoids. In February 2025, the patient experienced locoregional disease progression while maintaining excellent systemic disease control. Local radiotherapy was administered, and the patient achieved good disease control without the need for further therapy following discontinuation of immunotherapy, corresponding to a duration of response of approximately seven months.

## DISCUSSION

Poorly differentiated thyroid carcinoma is a rare and aggressive form of TC, with limited treatment options available in advanced stages. Our patient presented with locoregional invasion, pulmonary and osseous metastases, and a high tumor burden, necessitating prompt therapeutic intervention. Owing to a recent subarachnoid hemorrhage, standard systemic therapies such as tyrosine kinase inhibitors posed substantial risk, and radioiodine therapy was not indicated. In this context, pembrolizumab was considered based on a unique molecular profile suggestive of immunogenicity.

Systemic therapies for advanced TC, including sorafenib, lenvatinib, vandetanib, and cabozantinib, are associated with modest response rates and significant toxicity, particularly in poorly differentiated histologies. Specifically, in PDTC, sorafenib has demonstrated overall response rates of approximately 12%, while lenvatinib, cabozantinib, and vandetanib showed overall response rates between 30–35%, with hypertension and cardiovascular events occurring in up to 68% of patients ^(9-12)^. Given our patient’s recent cerebrovascular event, the risk-benefit profile of tyrosine kinase inhibitors was deemed unfavorable. Moreover, radioiodine therapy was not recommended, given the lack of iodine uptake in PDTC and the urgent need for systemic disease control.

DNA mismatch repair (MMR) defects can be identified by the loss of expression of any MMR protein (e.g., MSH2, MSH6, MLH1, and PMS2) using immunohistochemistry (IHC), indicating deficient MMR status (dMMR); or by polymerase chain reaction or NGS to detect microsatellite instability, defined as microsatellite instability-high (MSI-H). MMR alterations are present in approximately 2–4% of solid tumors and may be discordant in a minority of cases ^(13,14)^. Although MSI-H is the prototypical marker of dMMR, growing evidence suggests that dMMR can confer tumor immunogenicity independently of detectable MSI-H. This effect may result from the accumulation of point mutations and frameshifts at non-classical microsatellite loci that results in the formation of immunogenic neoantigens, and by virtue of the engagement of cytosolic nucleic acids activation of innate immune circuits, such as cGAS-STING. Additionally, current MSI testing strategies may underestimate genomic instability within a subset of dMMR tumors, thereby underrepresenting their true immunogenic potential. Collectively, these findings support the concept that MMR deficiency itself, rather than MSI status alone, may be sufficient to render tumors responsive to immune checkpoint blockade ^(15,16)^.

Both MMR deficiency and MSI-H are exceedingly rare in TC, a topography not typically associated with Lynch syndrome ^(13,14)^. In a series of 485 TC patients, including 17 cases of PDTC, only four were classified as MSI-H with dMMR, all of whom had follicular thyroid carcinomas. Notably, whole-exome sequencing of two MSI-H tumors in that cohort revealed a hemizygous loss-of-function *MSH2* mutation in one case ^(17)^. In a separate Korean study including 15 patients with PDTC, three exhibited high TMB (<13 mut/Mb), and one harbored a somatic *MSH2* mutation ^(18)^.

In our patient, molecular profiling revealed a frameshift mutation in *MSH2* and concurrent loss of *MSH2*/*MSH6* expression by IHC, with MSS on NGS. Although not MSI-H, the TMB of 10 mut/Mb exceeds the PDTC median of 2 mut/Mb and the 7.5 mut/Mb mean observed in PDTCs harboring MMR alterations ^(6)^. These findings suggest that *MSH2* loss may drive immunogenicity even in MSS tumors, highlighting a potential immune oncology-responsive to immune oncology therapies.

The TME of PDTC is characteristically immune-cold, with low PD-L1 expression and limited immune infiltration. Cameselle Garcia and cols. ^(8)^ evaluated the TME and PD-L1 status in 28 patients (15 with ATC and 13 with PDTC), finding that only one PDTC case surpassed the 1% TPS threshold by IHC ^(1)^. This outlier exhibited concurrent loss of *MLH1* and *PMS1*, a *TP53* mutation, and increased infiltration of CD3+CD8+ T lymphocytes, S100+ dendritic cells, and CD68+CD163+ macrophages, features characteristic of ATC. Our patient had a TPS of 20% in the context of an MSH2 mutation and dMMR/MSS status, suggesting convergence toward an ATC-like immune phenotype. This observation supports the hypothesis that MMR gene alterations may define a rare immunogenic molecular subgroup within PDTC ^(8)^.

Progressive dedifferentiation from WDTC to ATC occurs through the stepwise accumulation of molecular alterations ^(19)^. Mutations in *TP53* and *ATM* have been implicated in this process and are frequently associated with aggressive histologies. In PDTC, *ATM* mutations have been reported in approximately 7% of cases and up to 9% of ATCs, often co-occurring with *TP53* alterations ^(2,4)^. In our case, both mutations were present, supporting the interpretation that these genomic events may act as early drivers of dedifferentiation and underpin the clinically aggressive behavior observed, despite the absence of radioiodine refractoriness or previous systemic treatment. These findings also raise the possibility that such tumors may share therapeutic vulnerabilities with ATC.

Although clinical evidence supporting immune oncology in PDTC remains limited, it is mounting. In the phase 2 KEYNOTE-158 trial, pembrolizumab monotherapy produced objective responses in 7 out of 103 patients with advanced TC, including a complete response in one PDTC case. The median progression-free survival was 4.2 months ^(20)^. Another phase 2 trial evaluating ipilimumab plus nivolumab in aggressive TC included five PDTC cases, with a partial response in one patient ^(21)^. The ATLEP trial, which combined lenvatinib and pembrolizumab in 29 advanced TC cases, reported higher response rates in PDTC compared to ATC, regardless of PD-L1 expression ^(22)^. In our case, first-line pembrolizumab led to symptom resolution and a progression-free survival of approximately 7 months, exceeding historical controls and further supporting its potential role in biomarker-selected PDTC patients.

This report has some inherent limitations. First, pembrolizumab was administered as first-line therapy in a patient who had not received prior radioiodine therapy or tyrosine kinase inhibitors, limiting comparisons with standard therapeutic sequences. Second, although IHC confirmed the loss of *MSH2* and *MSH6*, we did not conduct additional analyses of tumor-infiltrating lymphocytes, regulatory T cells, or tumor-associated macrophages, which could have facilitated a more comprehensive characterization of the TME ^(6)^. Third, PDTCs harboring MMR gene mutations are exceptionally rare, which may introduce selection biases and limit the generalizability of findings from isolated reports. Nonetheless, this case possesses several strengths. It documents the use of pembrolizumab as upfront monotherapy in a molecularly defined PDTC with dMMR/MSS phenotype and intermediate TMB, resulting in rapid clinical improvement and a durable response. The report integrates genomic, pathological, and clinical data to bolster the rationale for checkpoint blockade in selected PDTCs, contributing valuable real-world evidence to a sparsely populated literature.

## CONCLUSION

In summary we present a rare case of metastatic PDTC with a confirmed dMMR phenotype characterized by MSH2 mutation and loss of MSH2/MSH6 protein expression despite an MSS profile. The tumor demonstrated intermediate TMB and a durable clinical response to pembrolizumab administered as first-line therapy. This case underscores the significance of comprehensive molecular profiling in thyroid carcinomas, particularly in identifying biomarkers beyond histologic subtype, such as dMMR by IHC or NGS. Our findings support the consideration of checkpoint inhibitors in selected PDTC patients, even in the absence of MSI-H. Further studies are warranted to clarify the predictive value of MMR alterations and TMB in the immunotherapeutic management of PDTC.

## Data Availability

all data generated or analyzed during this study are included in this published article.
